# Is the Mediterranean Low Fodmap Diet Effective in Managing Irritable Bowel Syndrome Symptoms and Gut Microbiota? An Innovative Research Protocol

**DOI:** 10.3390/nu16111592

**Published:** 2024-05-23

**Authors:** Arezina N. Kasti, Konstantinos Katsas, Konstantinos Petsis, Sophia Lambrinou, Kalliopi D. Synodinou, Aliki Kapetani, Kerry Louise Smart, Maroulla D. Nikolaki, Panagiotis Halvatsiotis, Konstantinos Triantafyllou, Jane G. Muir

**Affiliations:** 1 Department of Nutrition and Dietetics, Attikon University General Hospital, 12462 Athens, Greece; kastiare@med.uoa.gr (A.N.K.); katkonstantinos@gmail.com (K.K.); kostas.petsis@hotmail.com (K.P.); kall.synodinou@gmail.com (K.D.S.); akapetani@auth.gr (A.K.); kezbo2b@gmail.com (K.L.S.); maroullanikolaki@gmail.com (M.D.N.); 2 Medical School, National and Kapodistrian University of Athens, 75 Mikras Asias Street, 11527 Athens, Greece; 3 Department of Clinical Nutrition and Dietetics, General Hospital of Karpathos “Aghios Ioannis o Karpathios”, 85700 Karpathos, Greece; sophialambrinou@gmail.com; 4 Department of Nutrition and Dietetics Sciences, Hellenic Mediterranean University, 72300 Sitia, Greece; 5 2nd Propaedeutic Department of Internal Medicine, Attikon University General Hospital, Medical School, National and Kapodistrian University of Athens, 12462 Athens, Greece; pahalv@gmail.com; 6 Hepatogastroenterology Unit, 2nd Propaedeutic Department of Internal Medicine, Attikon University General Hospital, Medical School, National and Kapodistrian University of Athens, 12462 Athens, Greece; 7 Department of Gastroenterology, Monash University, Melbourne 3004, Australia; jane.muir@monash.edu

**Keywords:** irritable bowel syndrome, low FODMAP diet, Mediterranean diet, gut microbiota, SCFA

## Abstract

Introduction: Irritable bowel syndrome (IBS) symptoms can be effectively managed with the low FODMAP diet. However, its efficacy in reducing inflammation is not yet proven. On the contrary, the Mediterranean diet has anti-inflammatory properties with proven efficacy in treating chronic low-grade inflammation-related diseases. Aim: To publicly share our protocol evaluating the efficacy of the Mediterranean low-FODMAP (MED-LFD) versus NICE recommendations (British National Institute for Health and Care Excellence) diet in managing IBS symptoms and quality of life. Materials and Methods: Participants meeting the Rome IV criteria will be randomly assigned to MED-LFD or NICE recommendations and they will be followed for six months. Efficacy, symptom relief, quality of life and mental health will be assessed using validated questionnaires. In addition, fecal samples will be analyzed to assess gut microbiota, and to measure branched and short-chain fatty acids, and volatile organic compounds (metabolic byproducts from bacteria). Expected results and discussion: By publicly sharing this clinical study protocol, we aim to improve research quality in the field of IBS management by allowing for peer review feedback, preventing data manipulation, reducing redundant research efforts, mitigating publication bias, and empowering patient decision-making. We expect that this protocol will show that MED-LFD can effectively alleviate IBS symptoms and it will provide pathophysiology insights on its efficacy. The new dietary pattern that combines the LFD and the MED approaches allows for the observation of the synergistic action of both diets, with the MED’s anti-inflammatory and prebiotic properties enhancing the effects of the LFD while minimizing its limitations. Identifier in Clinical Trials: NCT03997708

## 1. Introduction

The Rome IV criteria highlighted a new insight into IBS’s pathophysiology, giving importance to the gut-brain axis interactions and to the low-grade inflammation restricted to the gut mucosa [1,2]. This low-grade mucosal inflammation, represented by the presence of mucosal enteroendocrine cells and T lymphocytes detected in rectal mucosa biopsy specimens and visceral hypersensitivity are present in all IBS subtypes, providing evidence that IBS has a structural component [3]. Research on the gut microbiota through a mega-cohort analysis revealed significantly lower α-diversity in IBS participants’ feces than in healthy controls, confirming that gut bacterial dysbiosis is associated with the above syndrome [4]. In a newly published review, gut microbiota in these patients showed reductions in *Bifidobacterium*, *Lactobacillus*, and *Faecalibacterium prausnitzii* relative abundances, increased levels of Firmicutes, and decreased *Bacteroidetes* (with an increased ratio of *Firmicutes*: *Bacteroidetes* ratio) at the phylum level [5]. *Lactobacillus* spp. and *Bifidobacterium* spp. are commensal bacteria that have probiotic properties and contribute to gut health [6]. Multiple meta-analyses have shown that probiotics have a positive effect on the symptoms of gastrointestinal ailments, including IBS [7], while *F. prausnitzii*, has anti-inflammatory functions that can also contribute to overall gut health [8]. They also identified several changes at lower taxonomic levels, including increased concentrations of *Clostridia* and *Clostridiales* [5]. *Clostridia* strains promote the release of IL-10, a protein that helps regulate the immune system. Seventeen bacterial strains from a healthy human fecal sample belonging to the *Clostridium* clusters IV, XIVa, and XVIII have been found to increase the number and function of colonic regulatory T cells in rodents. This is important because the *Clostridium* class is known to colonize the area around the colon’s mucosa, and it includes several major butyrate producers. These bacteria likely play a crucial role in regulating the host’s immune system. Additionally, some species within the *Clostridia* class are capable of producing biologically active catecholamines, such as norepinephrine and dopamine, which are neurotransmitters. This has been demonstrated in experiments with gnotobiotic and germ-free mice. Therefore, *Clostridia* seems to be particularly involved in the pathophysiology of IBS because of its important role not only in gastrointestinal immune homeostasis but also in the gut-brain axis [9]. On the other hand, the review detected decreased concentrations of *Bacteroidia* and *Bacteroidales*, while *Escherichia coli* and *Enterobacter* were found to increase in patients with gastrointestinal ailments compared to healthy controls [5]. *Bacteroides* are Gram-negative bacteria that help the host to obtain nutrients by breaking down food. However, in an unhealthy gastrointestinal environment, they can cause various endogenous infections and intraabdominal abscesses. Recently, they have also been associated with colon cancer. *Enterobacteriaceae* contain molecular components that directly enhance the inflammatory response, making them potentially harmful to gut health [10]. Environmental factors, like diet, are dominating in shaping the architecture of gut bacteria. Furthermore, up to 89% of IBS participants associate their symptoms with specific food consumption and 90% of them choose to eat less to reduce postprandial discomfort [3].

The low-FODMAP diet (LFD) is a well-documented intervention that reduces the symptoms of IBS and may represent the first-line treatment. The core mechanism of Fermentable oligo-, di-, monosaccharides, and polyols (FODMAPs) is related to their ability to attract water, which increases the volume of the fluid in the gut and contributes to its osmotic activity. Moreover, they provide food for bacterial populations, which ferment and produce gas [11].

On the other hand, the Mediterranean diet (MED) is mainly based on plant foods, with fish and poultry being the primary sources of animal products and a limited amount of dairy. Olive oil, being a significant product of the Mediterranean region, is also an essential component of this diet [12]. MED has antioxidant and anti-inflammatory properties that promote gut health through high amounts of phenolic compounds. Additionally, MED has been associated with an increased abundance of short-chain fatty acids (SCFAs) that help maintain a functional intestinal epithelium. Furthermore, olive oil, the primary source of fats in MED, has been linked to decreased intestinal inflammation and visceral hypersensitivity in animal studies. However, until recently, few studies have explored the impact of the MED on IBS symptoms [13,14]. In a recent study, a higher adherence to MED was associated with a lower abundance of potentially harmful *Faecalitalea, Streptococcus*, and *Intestinibacter*, and a higher abundance of potentially beneficial *Holdemanella* from the *Firmicutes* phylum [13].

In parallel, the nutritional recommendations of the British National Institute for Health and Care Excellence (NICE) for managing IBS represent some of the most commonly used guidance in clinical practice, based on dietary and lifestyle modifications [15].

Taking into consideration that LFD reduces IBS symptoms, but its efficacy regarding the reduction in inflammation is not proven, and that the MED is effective for chronic low-grade inflammation-related diseases, we are trying to determine the LFD’s benefits combined with the Mediterranean diets’ anti-inflammatory properties [15]. We aim to design and publicly share a randomized, open-labelled, placebo-controlled trial to evaluate the efficacy of the Mediterranean-LFD (MED-LFD) in comparison with the nutritional recommendations of the British National Institute for Health and Care Excellence for managing IBS symptoms.

## 2. Materials and Methods

### 2.1. Eligibility Criteria

Patients 18–65 years of age living in Greece who present with IBS symptoms will be assessed for fulfillment of the Rome IV diagnostic criteria for IBS diagnosis. Patients with Bristol Stool Form Scale (BSFS) types 3–7 with moderate to severe symptoms measured with IBS severity scoring system (IBS-SSS) > 175 will be enrolled. Full blood count, C reactive protein (CRP) or erythrocyte sedimentation rate (ESR), serum thyroid-stimulating hormone (TSH) within normal levels, negative results for tissue transglutaminase (tTg), immunoglobulin A or G antibodies (IgA or IgG) and negative fecal calprotectin are prerequisites for eligibility in the study. Participants > 50 years old should have completed colorectal cancer screening before enrollment.

### 2.2. Exclusion Criteria

Presence of (a) red flag symptoms, signs and/or laboratory findings indicating organic gastrointestinal disease like anemia, nocturnal diarrhea, gastrointestinal bleeding, unexplained weight loss, initiation of bowel symptoms after the age of 50, anorexia, systemic symptoms of inflammation like fever, persistent or deteriorating diarrhea and/or pain, fatigue, abnormal full blood count (FBC), high ESR or high CRP, (b) systemic disease requiring specialized diet, like cardiovascular disease, chronic renal failure, chronic liver disease or diabetes mellitus, celiac disease, cerebrovascular disease, major abdominal surgery, inflammatory bowel disease, (c) pregnancy, (d) breastfeeding, (e) IBS–constipation subtype (BSFS 1–2), (f) probiotic and antibiotic treatment within 1 month of day 1, (g) current hypnotherapy or cognitive behavior therapy, (h) history of food allergies, (i) inability to stop antispasmodic and/or antidiarrheal medications throughout the study period, (j) inability to follow the study requirements. Participants with an IBS-SSS score equal to or less than 175 will be also excluded.

### 2.3. Bioethics

The study will be conducted according to the Declaration of Helsinki following approval by the Institutional Ethics Committee of the University General Hospital “ATTIKON” (EBΔ435/19-06-2018). Written informed consent will be obtained from each participant following a detailed explanation of the objectives and protocol of the study.

This randomized clinical trial is registered under ClinicalTrials.gov, https://clinicaltrials.gov/ (accessed on 15 April 2024); Identifier: NCT03997708.

### 2.4. Interventions

MED-LFD and NICE diets will be provided to all participants by experienced dieticians in delivering LFD, following an initial consultation expected to last approximately 30 min.

#### 2.4.1. Randomization

We have prepared a random allocation sequence using block randomization, with a 1:1 ratio of control to intervention group (block size *n* = 4). Participants will be informed that both diets may reduce their symptoms but diet masking is impossible.

#### 2.4.2. Control Group

Participants in the control group will receive a weekly dietary plan for four weeks according to NICE guidelines for IBS participants [16], in which they may choose with free will foods from specific categories, i.e., vegetables, fruits, and grains. This includes regular meals, avoiding eating too little or too much, staying hydrated, reducing the intake of caffeine, alcohol, fizzy drinks, fatty, spicy, and processed foods, limiting fresh fruit intake to a maximum of three servings per day (approximately 80 g), high-fiber food (such as whole meal or high-fiber flour and bread, cereals high in bran, and whole grains such as brown rice), resistant starch, and sweeteners, and addressing any perceived food intolerances such as dairy [16]. An indicative analysis of the mean daily nutrition information of the NICE diet recommendation per week using https://explorefood.foodafactoflife.org.uk (accessed on 10 January 2024), is presented in Table 1.

#### 2.4.3. The MED-LFD Group

MED-LFD group (intervention) will receive a weekly dietary plan in line with their energy needs, adapted to the first phase (restrictive phase) of the strict LFD: the FODMAP content will not exceed 0.77–1.91 g per day (according to Monash University labs measurements, accessed on 21 October 2019). In this weekly plan, there is a free choice of animal protein, but participants will be strongly advised to consume fish 2–3 times/week and to reduce red meat to once per week. Furthermore, they will be guided to eat vegetables in small quantities, low in FODMADs, and to drink wine optionally, in the context of the Mediterranean way of life [17]. The components of the MED combined with LFD, acting synergistically without changing the initial profile of LFD, have been published in detail elsewhere [3]. An indicative analysis of the mean daily nutrition information of the restrictive phase of MED-LFD per week using https://explorefood.foodafactoflife.org.uk (accessed on 10 January 2024), is presented in Table 1.

##### Second Phase

Participants experiencing adequate relief symptoms during phase 1 will proceed to phase 2. During this phase (reintroduction), which will last for 6 to 8 weeks, participants will determine their tolerance to individual FODMAP subgroups such as fructose, lactose, galacto-oligosaccharides, fructans, and polyols. In this phase, we will find out which FODMAPs can be tolerated by introducing only one FODMAP subgroup at a time, typically for three days, with three washout days in between subgroups introductions [18]. At this stage, participants will be encouraged to use a diary to identify trigger foods and to record symptom responses to set filters in the Monash FODMAP app during the third phase. Details regarding the Monash FODMAP app are provided in Appendix A.

##### Third Phase

After completing the reintroduction phase, participants are expected to be able to identify the FODMAPs that trigger their symptoms and those that they can tolerate (personalization phase). Thereafter, they will follow a long-term diet that encourages nutritional adequacy, while they can customize the Monash FODMAP app’s food guide by setting filters that match their personal FODMAP sensitivities [14]. The personalized diet will include well-tolerated foods and FODMAPs, while poorly tolerated FODMAPs will be restricted to a level that provides adequate symptom control.

### 2.5. Consultations

After the initial consultation, participants will meet the same dietician on subsequent visits, physically or virtually. These visits are scheduled to occur up to four weeks after intervention initiation for the NICE group and six months after the first visit. In the MED-LFD group, visits are scheduled at the end of the restrictive phase to assist participants with implementing reintroduction, at the end of the reintroduction phase, and six months after the first visit.

### 2.6. Rescue Medication

Participants will be instructed not to take any medication that may influence IBS symptoms throughout the entire duration of the trial. However, in case of worsening symptoms, they are allowed to take painkillers such as paracetamol, paracetamol plus Hyoscine butyl bromide, antispasmodics, and loperamide, as rescue medication. All rescue medications will be recorded by the researchers.

### 2.7. Adverse Events, Serious Adverse Events, and Study Discontinuation

An adverse event (AE) is any undesirable or unfavorable medical occurrence in a participant or subject who is exposed to a medicine, a medical product, or an intervention like diet. An AE does not necessarily have a causal relationship with the medicine, product, or intervention. An AE can be any sign, symptom, or disease that is temporally associated with the use of the medicine, product, or intervention, whether or not considered related to it. An AE is serious and should be reported when the participant’s outcome is death, life-threatening, hospitalization, disability, or other significant impairment. All AEs will be recorded and causality association with treatment will be assessed. All serious adverse events (SAEs) will be reported according to the local legislation.

Participants can terminate their study participation at any time if they wish, or due to the occurrence of AE, progression, or intractable symptoms they are unable to control with the interventions and/or rescue medications. All discontinuations and their reason will be recorded.

### 2.8. Data Collection, Questionnaires and Evaluations

We will record demographic characteristics, such as age, sex, marital status, employment status, educational level, income and smoking habits from all subjects. Anthropometric data, such as weight, and height will be used to calculate the Body Mass Index (BMI) [weight (kg)/height^2^ (m^2^)] [19]. Participants with BMI < 18.5 kg/m^2^ are classified as underweight, 18.5 kg/m^2^ to <25 kg/m^2^ as normal, 25 kg/m^2^ to <30 kg/m^2^ as overweight and ≥30 kg/m^2^ as obese [20].

#### 2.8.1. Questionnaires

During the study periods, we will measure the severity of their symptoms using the IBS-SSS [21], which is validated in the Greek population [22], the general quality of life (QoL) using the 12-item Short Form Survey (SF12) questionnaire, which is commonly used [23,24] and validated in the Greek population [25], the IBS-associated quality of life using the Irritable Bowel Syndrome-Quality of Life Measure (IBS-QoL) [26,27], which has been validated in the past [28,29,30,31,32]. IBS burden symptoms will be measured using the Gastrointestinal Symptom Rating Scale-IBS version (GSRS-IBS) designed to assess gastrointestinal symptoms specifically for IBS participants [33]. The Hospital Anxiety and Depression Scale (HADS) questionnaire, validated in the Greek population [16], will be used to measure depression [HADS-D] and anxiety [HADS-A] separately [34]. Details regarding the aforementioned questionnaires are provided in Appendix B.

#### 2.8.2. Adequate Relief

Adequate relief (AR) is one of the most commonly used endpoint measures in IBS randomized controlled trials [35,36]. It will be evaluated with a single-item question, asking the participant: “In the last seven days, have you experienced adequate relief of your IBS symptoms?”. AR will be assessed through weekly telephone contact over four weeks for the NICE group and during the elimination reintroduction phases for the MED-LFD group. AR is achieved if positive answers are received in more than 50% of recordings within the given period. According to the study design, AR will be measured once after six months in both groups to evaluate symptom relief during the last week of follow-up.

#### 2.8.3. Adherence

Adherence to the diets will be assessed weekly by telephone, by asking the question: “How often did you follow your weekly diet?” and scored on a 5-point Likert scale (never, rarely, sometimes, most of the time, and every day).

### 2.9. Study Timepoints

**Figure 1:**
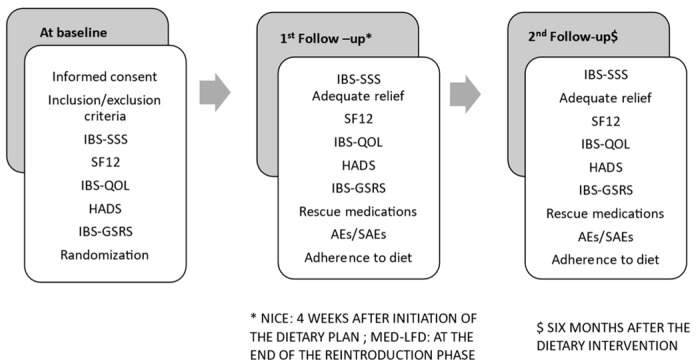
Study timepoints.

Three study timepoints are anticipated: Baseline: Two to four days before intervention initiation; first follow-up: at 4 weeks following the introduction of the dietary plan for the NICE group and at the end of the reintroduction phase for the MED-LFD group, anticipated to last on average 6–8 weeks (2–4 days’ time window); second follow-up: at six months (1-week time window), as shown in Figure 1, which also illustrates the procedures to be performed at each timepoint.

### 2.10. Study Endpoints

The study’s primary endpoint is the change from baseline of IBS-SSS at the first and the second follow-up. The co-primary endpoint is the rate of responders for each dietary intervention, defined by a ≥50-point reduction in IBS-SSS, which has been shown to be a clinically significant improvement [21,37], at the first and second follow-ups. Secondary endpoints measured at both follow-ups will include the rate of subjects reporting AR and changes in IBS-QoL, SF-12, HADS, and GSRS-IBS. Adherence to dietary intervention, use of rescue medications, rate of participants who achieve IBS-SSS < 175, discontinuation rate (and causes), and AE/SAEs comprise also secondary endpoints.

### 2.11. Statistics

#### 2.11.1. Sample Size Calculation

The sample size was calculated at 108 participants (54 per group) with expected values taken from the meta-analysis of Varju et al. [38] and a pre-specified statistical power of 80%, significance level α = 0.05, and 10% adjustment for non-adherence to detect a significant improvement in the primary outcome measure 100 vs. 59 points (estimated standard deviation = 60) in the MED-LFD and NICE groups, respectively.

#### 2.11.2. Study Populations

Intention-to-treat population (ITT) considers all randomized participants in the analysis, whether they complete the treatment as initially planned or not. Conversely, in the per-protocol (PP) analysis, we only analyze data from those who strictly adhere to the study protocol [39]. Responders (defined as participants with at least a ≥50-point reduction in IBS-SSS), AR rates, and the rate of participants who achieve IBS-SSS < 175 will be measured in the ITT and PP population; IBS-SSS, IBS-QoL, SF-12, HADS, GSRS-IBS change and use of rescue medications will be measured in the PP population. Discontinuation rate (and causes) and AE/SAEs will be measured in the ITT population.

#### 2.11.3. Statistical Analysis

Categorical variables will be presented as relative frequencies (%) and continuous variables as mean values (standard deviation) for those normally distributed and as median (interquartile range) for those not normally distributed. Normality will be evaluated graphically (i.e., using histograms, p-p plots, boxplots, q-q plots) and statistically through the Kolmogorov Smirnoff test. Student’s *t*-test will be used to examine differences in all quantitative variables and chi-squared test for all categorical variables between the two intervention groups (NICE vs. MED-LFD). Paired analysis will be utilized to compare all scores between baseline, first follow-up and second follow-up, independently, and also a test-for-trend will be performed to assess any potential longitudinal changes. For the data analysis, the statistical package STATA 16 will be used, and a *p*-value of ≤0.05 will be regarded as statistically significant.

### 2.12. Exploratory Analyses

Aiming to explore the physiology mechanism implicated in the development of the positive effects of the MED-LFD in IBS symptoms management, we foresee the following mechanistic exploratory analyses in a subgroup of 40 participants (20 from each intervention group) who will provide stool samples at each study timepoint for gut microbiota analysis using metagenomics, and short-chain fatty acids (SCFAs)—acetate, butyrate, and propionate—the intestinal bacterial metabolites produced by fermented nondigestible carbohydrates [11], measurement using Gas Chromatography/Mass Spectrometry. In this subgroup, detailed measurements of the adherence to the diet will be assessed with three-day 24 h dietary recalls (on consecutive days, including a weekend day) twice by telephone contact. The first one at two weeks after interventions started, and the second six months after interventions started for both groups. The dietary recalls aim to calculate the number of consumed FODMAPs per day.

#### 2.12.1. Fecal Samples Collection

Following signing specific informed consent, participants will be asked to provide a stool sample at three different timepoints: baseline, first follow-up, and second follow-up. The stool samples will be immediately frozen after collection and stored at −80 °C in 2 mL screw cap cryovials until further analyses.

#### 2.12.2. Metagenomic Analysis

The analysis will be undertaken to evaluate the effects (immediate and long term) of the two diets on the participants’ microbiome. The exact protocol for the metagenomic analysis is shown in Appendix C.

#### 2.12.3. Short-Chain Fatty Acids Measurements

Using Gas Chromatography/Mass Spectrometry (GC/MS), gaseous eluted compounds like the short-chain fatty acids in the stool are ionized, their ions are separated based on their mass-to-charge ratios, and the intensity of each ion is measured [40]. We aim to investigate the changes in the fecal short-chain fatty acids intensity (immediate and long term) and to compare them between the two IBS groups. The exact protocol for the GC/MS analysis is shown in Appendix D.

## 3. Expected Results and Discussion

During the last two decades, campaigns initiated by impact journals have advocated for access to original trial data, including the case record forms [41]. Sharing clinical study protocols can significantly enhance the caliber of research through various means: It permits researchers to receive input on their protocols via peer review, although studies with ethical approval or funding often receive feedback on initial drafts. However, even the most meticulously planned study might encounter challenges defending contentious elements in its protocol. Moreover, the publication of protocols facilitates comparisons between the original intentions and the actual execution, crucial for preventing data manipulation or selective reporting. Making study protocols public helps researchers comprehend ongoing studies, curbing redundant research efforts. It also assists systematic reviewers in identifying trials, potentially mitigating publication bias. Lastly, the disclosure of study protocols allows participants to identify studies they might consider participating in, empowering their decision-making process [42].

We present herein our single-center, randomized controlled study to evaluate the clinical effects of the LFD combined with the elements of the MΕD in comparison to the dietetic consultations of the NICE for the management of IBS symptoms. Additionally, we foresee the conduction of two physiology studies aiming to better understand the clinical results of the study.

Chen et al. found that while standard MΕD was not associated with the severity of the disease, certain MED foods were linked to symptoms indicating personalized management for those with severe symptoms [13]. Paduano et al. concluded that MΕD is well-accepted and easier to follow than other proposed diets for short-term periods (4 weeks), while they did not find evidence of LFD superiority against MED in improving the quality of life of IBS participants [14]. Moreover, a diet that excludes nutrient-rich foods can lead to nutritional inadequacy. Introducing LFD for a long period can result in insufficient intake of essential nutrients like carbohydrates, fiber, iron, folate, magnesium, vitamin C, B vitamins, and calcium [43,44,45].

According to NICE guidelines, specific lifestyle changes may improve IBS symptoms. It is important to note that FODMAP-containing foods are not specifically restricted in the guidance, but it may be helpful to limit the intake of high-fiber foods [46]. The same dietary advice is followed in the restrictive phase of LFD where fiber consumption is restricted [3,43]. However, dietary fiber increases the levels of SCFAs such as acetate, propionate, and butyrate in the colon through gut microbial fermentation [47], which pose anti-inflammatory properties, enhance the immune response, and contribute to gut barrier integrity. SCFAs have been proven to improve gut health through several local effects, such as maintaining intestinal barrier integrity, mucus production, and protection against inflammation, as well as reducing the risk of colorectal cancer [48]. Despite the challenging nature of IBS, diet and dietary end-products are linked to both the development and management of the syndrome. While most of the literature focuses on SCFAs, research on how BCFAs influence the dietary management of IBS is limited. Nevertheless, LFD is an established dietary intervention that has been found to alleviate IBS symptoms. It leads to higher protein and amino acid fermentation, and, as a result, may produce BCFAs, the byproduct of this fermentation. Despite the protective role that BCFAs might play in gut inflammation [11], other nitrogenous metabolites of protein fermentation, such as amines, hydrogen sulfide, p-cresol, phenols, and ammonia, have been found to have detrimental effects on colonocytes and are associated with gut inflammation, a condition that has been pathogenetically linked with IBS, and consequently with quality of life. In addition, reduced diversity of the gut microbiota and the presence of *Clostridiales*, *Prevotella*, and methanogenic species were proposed as an IBS-specific microbiome signature that is associated with the severity of symptoms [49]. These properties are essential for IBS, which is characterized by increased intestinal permeability, intestinal dysbiosis, and low-grade inflammation [11,50].

According to our study protocol, both groups are expected to consume an average of 25 g of fiber daily. While the literature provides insufficient data to determine the optimal dosage or duration of fiber consumption, the American Academy of Nutrition and Dietetics recommends a daily fiber intake of 25 g for women and 38 g for men, which can benefit IBS participants of all subtypes and improve overall well-being [51].

This trial introduces a new dietary pattern that combines the LFD and the MED to manage IBS symptoms. The innovative aspect of this approach lies in the synergistic action of both diets, with the MED’s anti-inflammatory and prebiotic properties enhancing the effects of the LFD while minimizing its limitations. We believe that enriching the LFD with the components of the MED will effectively optimize its benefits and alleviate the symptoms of IBS. Moreover, the two exploratory analyses are expected to provide physiological grounds to explain the clinical results.

Our study protocol has limitations. One of these is the lack of blinding; this will affect the clinical response to some extent. Additionally, we are unable to examine the effects of systematically altering dietary fiber intake in IBS patients because we do not differentiate between soluble and insoluble dietary fibers. A serious limitation for a trial that wishes to prove the advantages of a Mediterranean pattern compared to the comparator is that both groups will have similar access to extra virgin olive oil intake—the primary source of fat in Greek cuisine—and its anti-inflammatory properties benefitting the entire IBS cohort [51]. Another limitation is that some methodologies appear resource-intensive, involving sophisticated equipment and specialized reagents (e.g., Nanopore, GC/MS). Although such resource requirements could pose challenges for replication and broader implementation in settings with limited access to these resources, these are referred to as exploratory analyses to understand more clearly the pathophysiology dietary manipulation on microbiota and on BCFAs/SCFAs production in IBS. While potential changes in gut microbiota composition may be notable, a critical assessment requires considering various factors such as study design, context, causality, functional implications, and the complexity of the gut microbial ecosystem. Further research and a nuanced approach might be necessary to draw meaningful conclusions about the implications of these microbiota changes in different health conditions.

## 4. Conclusions

Changing a patient’s diet is a daily activity, thus making long-term dietary changes potentially preferable to expensive drug therapy. Therefore, a diet that restricts the consumption of trigger foods or one that corrects gut microbiota imbalances could help manage IBS symptoms. In the literature, the LFD appears to positively influence the symptom’s severity, relieving abdominal pain, bloating, and diarrhea [46]. The novelty of this clinical trial is the concept of a new dietary pattern with the synergistic action of the LFD and MedDiet as a dietary treatment for IBS. The long-term effects of the LFD on the interaction between the gut epithelium and microbiota have not been fully established. Conversely, a diet rich in nutrients with anti-inflammatory properties, such as the MD, may be associated with improved immune function and reduced inflammation. Dietary recommendations should not only focus on improving IBS symptoms but also on enhancing quality of life. We hope that the clinical outcome measures of our study will shed light on the specific research questions described above and that the explanatory analysis to be undertaken will give insights on the pathophysiology of the intervention.

## Figures and Tables

**Table 1:** Indicative analysis of the mean daily nutrition information of each treatment group.

	NICE Group	MED-LFD Group
Energy intake	1797 (kcal)/7585 (kJ)	1799 (kcal)/7527 (kJ)
Proteins (g)	86.53	85
Fats (g)	61.68	80
Saturated (g)	14.39	16.9
Monounsaturated (g)	27.64	43.6
Polyunsaturated (g)	6.86	13.3
Carbohydrates (g)	239.79	197
Total Sugars (g)	101.08	59.1
Fiber (g)	25.27	24.95
* Total FODMAPs (g)	-	1.25
* Oligosaccharides (g)	-	1.22
* Polyols (g)	-	0.23
* Lactose (g)	-	0.11
* Galactooligosaccharides (g)	-	0.03
* Fructose in excess of glucose (g)	-	0.48
* Fructans (g)	-	1.29

* Total FODMAPS per day according to Monash University labs. Data retrieved from: https://explorefood.foodafactoflife.org.uk (accessed on 10 January 2024).

## Data Availability

The data presented in this study are available on request from the corresponding author, due to privacy and ethical reasons.

## Abbreviations

AE	Adverse events
AR	Adequate relief
BMI	Body Mass Index
BSFS	Bristol Stool Form Scale
BSTFA	Bis(trimethylsilyl)trifluoroacetamide
DE	Diethyl Ether
FODMAP	Fermentable oligo-, di-, monosaccharides and polyols
GC/MS	Gas Chromatography/Mass Spectrometry
GSRS-IBS	Gastrointestinal Symptom Rating Scale-IBS version
HADS	Hospital Anxiety and Depression Scale
HADS-D	The HADS questionnaire measures depression
HADS-A	The HADS questionnaire measures anxiety
IBS	Irritable Bowel Syndrome
IBS-SSS	IBS severity scoring system
ITT	Intention-to-treat population
QoL	Quality of Life
LFD	Low FODMAP Diet
MED	Mediterranean Diet
MED-LFD	Mediterranean Diet-Low FODMAP Diet
MCS	Mental Composite Score
NICE	National Institute for Health and Care Excellence
PCS	Physical Composite Score
PCR	Polymerase Chain Reaction
PP	Per Protocol
SAEs	Serious Adverse Events
SF12	12-Item Short Form Survey
SCFAs	Short-chain Chain Fatty Acids

## Appendix A. Monash FODMAP Diet App

The Monash FODMAP diet app contains the largest database of FODMAP foods in the world. The food guide is based on the ‘traffic light system’ to simplify the FODMAP content of foods. “Green light” indicates safe low-FODMAP foods, “orange” foods that should be consumed in moderation, and “red light” indicates a high level of FODMAPs, while also providing detailed information about which FODMAP(s) contains each food. Certain foods can be high, moderate, or low in FODMAPs, depending on how much is eaten. Food ranking is also affected by portion size. The filters help participants adjust the food guide to their intolerances. After setting the filters, searching for individual foods or skimming one of the eight food group lists will include a green, yellow, or red light, or will be greyed out completely [52].

## Appendix B. Questionnaires

### Appendix B.1. IBS-SSS

The IBS-SSS questionnaire consists of five specific questions [21] and it has been recently validated in a Greek cohort of patients [22]. Each of the five questions (pain severity, pain frequency, abdominal distension severity, bowel movement satisfaction, quality of life) ranges from a minimum score of 0 to a maximum score of 100, using a visual analog scale (VAS), with total scores ranging from 0 to 500. Higher scores indicate more severe symptoms. Subjects can be categorized as having mild (75–175), moderate (175–300), or severe (>300) IBS symptoms. An IBS-SSS decrease by 50 or more points is considered as representing significant symptoms improvement [21].

### Appendix B.2. Twelve (12)-Item Short Form Survey (SF12)

SF-12 is a 12-item questionnaire used to assess generic health outcomes from the participant’s perspective, covering two domains of health outcomes: physical functioning and mental health [23]. The items are weighted and summed to provide physical and mental health scores (PCS and MCS). The two composite scores are computed using the scores on the questions that range from 0 to 100, with a higher score indicating better QoL.

### Appendix B.3. Irritable Bowel Syndrome-Quality of Life Measure (IBS-QoL)

The IBS-QoL assesses the degree to which IBS interfered with quality of life over the past 30 days [26]. It is a self-reported quality of life measure, which includes eight domains, such as dysphoria, interference with activity, body image, health worry, food avoidance, social relations, sexual and relationship issues. Each of them is rated based on a 5-point Likert scale (1. “Not at all”; 2. “Slightly”; 3. “Moderately”; 4. “Quite a bit” 5. “Extremely/A great deal”). All questions are reversed and summed using a transformation formula (see “Information Sheet on the IBS-QOL” [https://depts.washington.edu/seaqol/docs/IBS-QOL_Info.pdf (accessed on 20 April 2024)], providing a total score from 0% to 100%, with a higher score indicating better QoL. It measures the extent to which IBS has affected the quality of life over the past 30 days, and includes eight domains: dysphoria, interference with activity, body image, health worry, food avoidance, social relations, and sexual and relationship issues. The questionnaire provides a total score ranging from 0% to 100%, where a higher score indicates a better quality of life.

### Appendix B.4. Gastrointestinal Symptom Rating Scale-IBS Version (GSRS-IBS)

The GSRS-IBS is used to assess the symptoms burden in IBS participants. The GSRS questionnaire was originally developed for dyspeptic participants but was later validated in participants with IBS. GSRS-IBS consists of 13 items, designed to assess gastrointestinal symptoms specifically for IBS participants [33]. Each item is rated based on a 7-point Likert scale (1. “No discomfort at all”; 2. “Minor discomfort”; 3. “Mild discomfort”; 4. “Moderate discomfort”; 5. “Moderately severe discomfort”; 6. “Severe discomfort”; 7. “Very severe discomfort”). The questionnaire has a total score and five subscales (pain, bloating, constipation, diarrhea and satiety), calculated by summing all questions corresponding to each scale and dividing with the number of questions summed in each scale. Both total and each subscale scores are ranging from 1 (no discomfort) to 7 (severe discomfort), with higher scores representing increased symptom burden.

### Appendix B.5. Hospital Anxiety and Depression Scale (HADS)

HADS questionnaire is used for the assessment of anxiety and depression symptoms [34], and it is validated in the Greek population [16]. It is a 14-item questionnaire, consisting of two different subscales (depression [HADS-D] and anxiety [HADS-D] subscale). Each subscale includes seven items, each scored on a response scale with four alternatives, ranging between 0 and 3. The total score is the sum of the 14 items, and for each subscale, the score is the sum of the respective seven items (scores range from 0 to 21). Participants may be classified in each subscale based on their scores as ‘normal’ (scores 0–7), ‘borderline abnormal’ (scores 8–10) or ‘abnormal’ (scores 11–21).

## Appendix C. Laboratory Steps of the Metagenomics Analysis

### Appendix C.1. DNA Extraction from Stool Samples

Total DNA is purified from stool samples utilizing the QIAamp Fast DNA Stool Mini Kit (Qiagen, Hilden, Germany). In summary, fecal samples weighing between 180 and 220 mg are added to 2 mL microcentrifuge tubes and kept on ice. To each fecal sample, 1 mL InhibitEX Buffer (Qiagen, Hilden, Germany) is added, and the tubes are vortexed constantly for 1 min to ensure thorough homogenization. Samples are then spun at full speed for 1 min to form a pellet of stool particles. Further steps are completed following the manufacturer’s instructions for the Isolation of DNA from Stool for Human DNA Analysis protocol. The extracted DNA samples are preserved at −20 °C until further analysis.

### Appendix C.2. Gut Microbiota Analysis Using Oxford Nanopore Technologies

We will use third-generation sequencing to determine DNA sequences. Specifically, we will investigate the extracted DNA by 16S rRNA gene sequencing using the portable handheld “MinION” device from Oxford Nanopore Technologies, Oxford, UK. Through nanopore sequencers, we will generate native DNA sequences from stool samples rapidly, from which each long-read is called in real-time to provide analyses such as taxonomic classification through the built-in software.

This method is based on the fact that the conductivity of the pore changes when a clone of nucleic acid passes through it. The ion current flow is based on the displacement of the molecule within the pore. The electrolyte ions in the solution move through the pore under a predetermined voltage, creating an ionic current signal [53]. When a molecule, such as a negatively charged DNA molecule is added to the solution, the current flowing through the nanopore will be blocked, interrupting the transmitted signal. The physical and chemical properties of the target molecules can be calculated by statistical analysis of the amplitude and duration of the currents [54]. Each nucleotide provides a unique electrical signature determined by the orientation and load properties [55]. Nanopore sequencing will create a single-use DNA sequencer, the size of a USB memory stick, which will be designed for general applications of DNA sequencing. The average read length of MinION is about 5.4 kb to 10 kb which is much longer than the average read lengths of other sequencing technologies [56].

The protocol integrates enzymatic lysis steps with DNA purification and size-selection approaches. Requiring < 1 g of input sample, our approach can yield microgram quantities of output DNA with fragment peak lengths in the tens of kilobases. This yield is sufficient for direct use with the Oxford Nanopore sequencing, without the need for whole-genome amplification or PCR-based amplification approaches.

The following steps should be taken for library preparation and sequencing using the Oxford Nanopore Genomic DNA by Ligation library preparation kit: DNA repair, DNA end preparation, and sequencing adapter attachment. To ensure maximum yield and an optimized ratio of available DNA ends to sequencing adapters, the input DNA should be adjusted based on the peak size and total mass to 100–200 fmol, as instructed in the extended Genomic DNA by Ligation protocol. AMPure bead cleanup steps should also be incorporated to improve sample purity before sample loading. The Oxford Nanopore Rapid Sequencing protocol is an acceptable alternative for DNA extractions with lower total mass. This protocol can be performed in just 10 min and uses transposome-mediated tagmentation to attach sequencing adapters to DNA. The suggested input for this protocol is 400 ng [57].

## Appendix D. Short-Chain Fatty Acids Measurements

### Appendix D.1. Gas Chromatography/Mass Spectrometry Analysis

In Gas Chromatography/Mass Spectrometry (GC/MS), the gaseous eluted compounds are ionized, their ions are separated based on their mass-to-charge ratios, and the intensity of each ion is measured [40]. We will use a QP-2010 Ultra by Shimadzu Inc., Kyoto, Japan, with a BPX-5 capillary column with 30 m × 0.25 mm dimensions and 0.25 μm film thickness for GC/MS analysis. To increase the sensitivity and avoid contamination, we will use N, O-bis(trimethylsilyl)trifluoroacetamide (BSTFA) as a derivatization reagent and anhydrous sodium sulfate (Na_2_SO_4_) as an alternative moisture removal method [58]. The injector, ion source, quadrupole, and the GC/MS interface temperature will be set to 260, 230, 150, and 280 °C, respectively.

The carrier gas (helium) transports the compounds through the column and to the detector. We will inject 1 µL of the derivatized sample with a 3 min solvent delay time and a split ratio of 10:1. The column temperature is initially set to 40 °C for 2 min, then increased to 150 °C at a rate of 15 °C/min and stabilized for 1 min, until it finally reaches 300 °C at a rate of 30 °C/min and it is maintained this temperature for 5 min. We will carry out the ionization in the electron impact mode at 70 eV. The analytes will be quantified in the selected ion monitoring mode using the target ion and confirmed by confirmative ions [58].

### Appendix D.2. SCFAs Extraction

Short-chain fatty acids (acetate, butyrate, and propionate) are intestinal bacterial metabolites produced by fermented nondigestible carbohydrates [11].

Their extraction process involves several stages. The first step is to mix 30 mg of stools with 300 µL of pure water and homogenize the mixture for 20 s under 6500 rpm (rounds per minute), repeating the process three times using a Precelly 24 homogenizer from Bertin Technologies, Montigmy le Bretonnexux, France. The next step is to incubate the samples at 4 °C with shaking for 30 min, followed by centrifugation for 30 min at 13,000× *g*. After centrifugation, 100 µL of supernatant is transferred into a new 0.6 mL microtube that has 10 µL of 5 M HCl added to it, which brings the pH of the fecal solution to 2. The acidified stool samples homogenate is extracted by adding 100 µL of anhydrous diethyl ether (DE) (1:1, *v*/*v*), vortexing and incubating on ice for 5 min, and then centrifuging for 5 min at 10,000× *g*. The DE layer containing SCFAs is then transferred to a new microtube containing anhydrous Na_2_SO_4_ to remove residual water. The remaining aqueous layer is further extracted with DE two more times. The DE layers are pooled and mixed for further derivatization.

The process of derivatization involves transferring 100 µL of the DE extract into a glass insert in a GC vial after adding 5 µL of BSTFA and vortexing for 5 s. The mixture is then kept in the GC vial and incubated at 70 °C for 20–40 min, followed by 37 °C for 2 h. The derivatized samples are then loaded onto GC/MS. To correct the background, pure water is used as a blank sample, which is processed with the same procedure as that of fecal samples [58].
